# Can a ‘rewards-for-exercise app’ increase physical activity, subjective well-being and sleep quality? An open-label single-arm trial among university staff with low to moderate physical activity levels

**DOI:** 10.1186/s12889-021-10794-w

**Published:** 2021-04-23

**Authors:** Sakari Lemola, Anna Gkiouleka, Brieze Read, Anu Realo, Lukasz Walasek, Nicole K. Y. Tang, Mark T. Elliott

**Affiliations:** 1 Department of Psychology, University of Warwick, University Road, Coventry, CV4 7AL UK; 2 Department of Psychology, Bielefeld University, Bielefeld, Germany; 3 Department of Public Health & Primary Care, University of Cambridge, Cambridge, UK; 4 Institute of Digital Healthcare, WMG, University of Warwick, Coventry, UK; 5 Institute of Psychology, University of Tartu, Tartu, Estonia

**Keywords:** Mobile applications, Physical activity, Subjective well-being, Sleep quality, Behaviour change, Extrinsic incentives

## Abstract

**Background:**

This study examined the impact of a ‘rewards-for-exercise’ mobile application on physical activity, subjective well-being and sleep quality among 148 employees in a UK university with low to moderate physical activity levels.

**Methods:**

A three-month open-label single-arm trial with a one-year follow-up after the end of the trial. Participants used the Sweatcoin application which converted their outdoor steps into a virtual currency used for the purchase of products available at the university campus’ outlets, using an in-app marketplace. The primary outcome measure was self-reported physical activity. Secondary measures included device-measured physical activity, subjective well-being (i.e., life satisfaction, positive affect, negative affect), and self-reported sleep quality.

**Results:**

The findings show an increase in self-reported physical activity (d = 0.34), life satisfaction (d = 0.31), positive affect (d = 0.29), and sleep quality (d = 0.22) during the three-month trial period.

**Conclusion:**

The study suggests that mobile incentives-for-exercise applications might increase physical activity levels, positive affect, and sleep quality, at least in the short term. The observed changes were not sustained 12 months after the end of the trial.

**Supplementary Information:**

The online version contains supplementary material available at 10.1186/s12889-021-10794-w.

## Background

Physical activity is associated with better health and well-being [3, 6, 8, 18, 31, 39, 42]. Regular physical exercise reduces systolic and diastolic blood pressure among healthy adults [11], improves health indicators in chronic patients [46], and is known for its therapeutic effects on mental health, particularly on reducing depressive symptoms [10]. Individuals who follow the WHO recommendation of 150 min of moderate aerobic activity per week [52] show reduced day-time (e.g. sleepiness) and night-time symptoms (e.g., difficulty in falling asleep) of insomnia [21] and better sleep quality [26], although the extent of such benefits varies according to age and sex (see for example [7, 32]). Moreover, increased physical activity among adults is associated with higher levels of both affective and cognitive aspects of subjective well-being (SWB [27, 42];). For example, it has been found that routine activities like walking have a positive impact on mood [24] and that leisure time exercise has a positive effect on perceived quality of life mediated via increased positive and decreased negative affect [23].

Walking at a pace of 5 km/h meets the definition of moderately intense activity [36] and has been described as a form of exercise to develop and sustain physical fitness [4, 34]. Still, initiating and maintaining suitable levels of physical activity remains a challenge for many people. A key driver of physical inactivity is that people are often physically inactive at work, with a large proportion of the population in desk-based jobs that involve long periods of sitting [25, 50]. Therefore, identifying strategies to increase levels of physical activity at a population level is a major public health priority [37]. It has also been suggested that employers might be interested in encouraging physical activity of their staff as active employees may not only be healthier but also show better performance at work [29].

The advent of smartphone and wearable technologies has provided a platform for innovative approaches that motivate activity [19, 40, 43–45]. A specific type of smartphone application aims to increase physical activity through extrinsic incentives and rewards [22]. One such application is Sweatcoin, which records the number of outdoor steps and converts them into a virtual currency that can be used to purchase goods and services from the in-app public marketplace [14]. In addition, Sweatcoin offers an opportunity to maintain a physical activity profile and to interact and compare activity patterns with other users. A study on 5892 young adult Sweatcoin users showed an average 18.7% increase in daily step count over 6 months following registration with the app, compared to a 3-month period before registration [15].

Such findings are consistent with research showing that financial incentives may encourage physical activity [2, 17, 30]. However, while apps and wearables can improve levels of physical activity [19, 40, 45], existing evidence shows that changes are usually not sustained in the long run (i.e. no longer than 3 months) [43]. Further, there is a lack of evidence regarding whether effects of mobile Health (mHealth) applications may spill over to other health related domains and for instance also increase SWB and sleep quality.

The present study examines whether the use of the Sweatcoin application in a work environment increases physical activity levels among physically ‘inactive’ to ‘moderately active’ employees, and whether this further leads to wider improvements in their sleep quality and SWB. Earlier research has suggested that when examining the associations between SWB and health-related variables, the components of SWB should be assessed as distinct constructs [41] and for this reason, we examined three components of SWB—life satisfaction, positive affect and negative affect—separately in the following analyses, so that we would not lose any valuable information about SWB by merging them (cf. [13]).

We conducted a 3-month open-label single-arm trial with university staff, where participants could use the in-app public marketplace in the regular way with additional access to a ‘local marketplace’ including products from university outlets. Twelve months after the intervention involving access to the ‘local marketplace’ had ended, the trial was followed-up by a survey to examine whether potential changes were sustained in the long-term.

The following hypotheses were tested: *H1a*: Participants’ self-reported and device-measured physical activity increases from before the use of the Sweatcoin application to 3 months of consecutive application use; and *H1b:* the observed increase in physical activity is sustained until 12 months after the end of the trial. *H2a*: Participants’ levels of SWB and sleep quality increase from before the use of the Sweatcoin application to 3 months after consecutive application use; and *H2b*: the observed increase in SWB and sleep quality is sustained until 12 months after the end of the trial.

The hypotheses and associated analyses were registered prior to the data analysis [AsPredicted No: 19317 https://aspredicted.org/v38eh.pdf].

## Methods

### Study design and the ‘Sweatcoin’ application

**Fig. 1:**
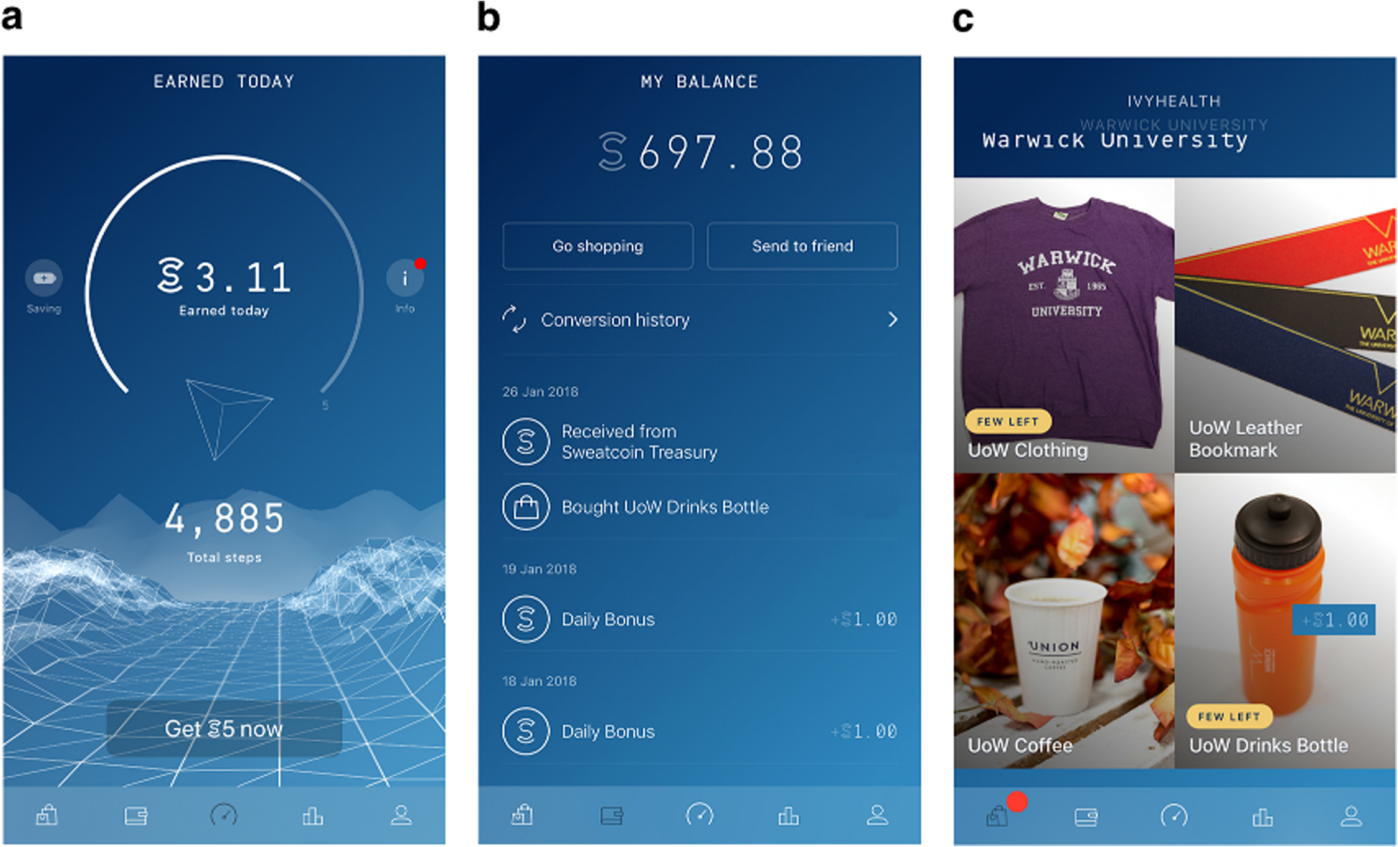
Example screenshots from the customised Sweatcoin application. Participants’ daily step count was captured by the app before being verified (only outdoor steps were rewarded) and converted to Sweatcoins (**a**). Accumulated coins are stored in the digital wallet along with a record of transactions (**b**). Sweatcoins can be used to buy products on an in-app marketplace; here a local marketplace was added to the app, where participants could be products available from campus retail outlets (**c**). Permission to publish the images was obtained from Sweatco Ltd. (Copyright holder)

The study involved an opportunity sample of staff members (both academic and administrative and support staff) at the University of Warwick (Coventry, UK). Staff were recruited via a University newsletter emailed to all staff, and through digital signage presented on screens distributed across University buildings. The single-arm open-label trial took place between February and May 2018, with a follow-up survey 12 months after the intervention had ended (May 2019). The study received ethical approval by the University of Warwick Biomedical and Scientific Research Ethics Committee (BSREC) (Reference number: REGO-2017-2070). At the beginning of the trial, participants who met the inclusion criteria (see below: Data and Participants) were invited to download a special version of the ‘Sweatcoin’ app to their smartphone. The application converted participants’ outdoor steps into a virtual currency at a rate of 0.95 Sweatcoins per 1000 verified steps (Fig. 1). Due to the application using a verification algorithm that relies on Global Positioning System (GPS) signals to reward genuine steps, only outdoor steps were converted. Accumulated coins could be used to purchase real products via an in-app marketplace. The only difference between the customised app and the publicly available version was that besides the in-app public marketplace, the former included also a ‘local marketplace’ with products available from retail outlets in the University campus (see Supplementary Table 1).

### Data and participants

#### Participants’ recruitment

Two-hundred and fifty-three (253) university staff members (both academic as well as administrative and support staff) registered their interest in the study and completed an initial screening questionnaire used to check the eligibility to participate according to the following inclusion criteria^1^:

- They were using the Sweatcoin app for the first time (excluded *n* = 13 who had been using the app before).
- They were deemed ‘moderately active’ or ‘inactive’ as determined by the General Practice Physical Activity Questionnaire (GPPAQ [38];; score less than 3) (excluded *n* = 48 who were more than moderately active).
- They owned a compatible smartphone (either Apple iPhone 5S or above or an Android-based phone running v4.4 KitKat or above) (none excluded)
- They were older than 18 years and younger than 70 years of age (none excluded).

**Fig. 2:**
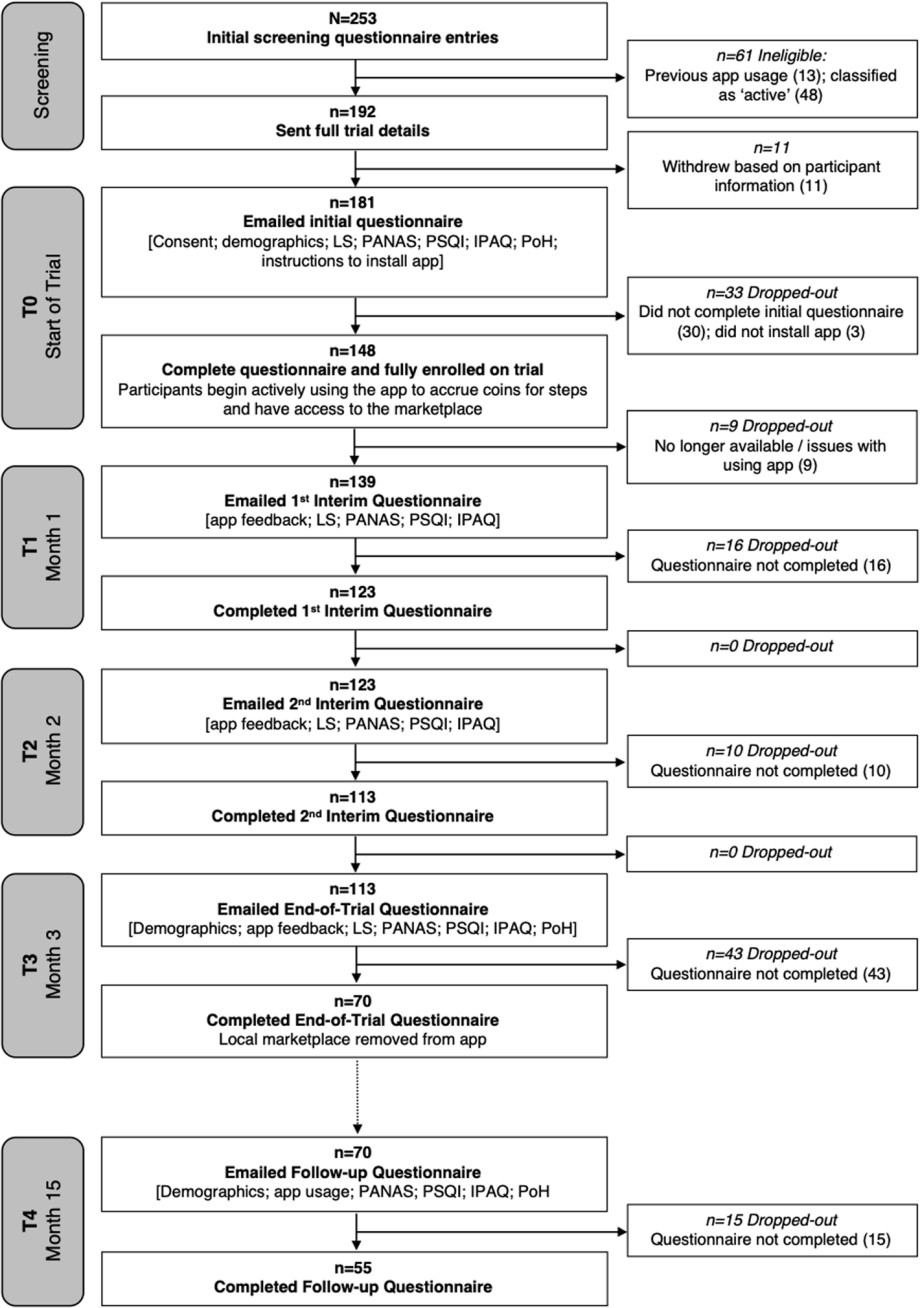
An overview of participant enrolment and drop-out as the trial progressed. Abbreviations: IPAQ: International Physical Activity Questionnaire [12]; LS: Life satisfaction questions; PANAS: Positive and Negative Affect Survey [51]; PoH: Perception of Health questions; PSQI: Pittsburgh Sleep Quality Index) [5]

One-hundred and ninety-two (192) members of staff received an information leaflet, of which 11 withdrew citing reasons such as *“not using their phone frequently”* or *“not wishing to leave their GPS on all day”*. One-hundred and eighty-one (181) participants received the initial questionnaire (T0) and 151 of them completed it. Three participants failed to download the app, resulting in 148 participants fully enrolled in the study. Subsequently, nine more participants withdrew due to non-availability or reluctance. One-hundred and thirty-nine (139) participants subsequently received the first interim-questionnaire (T1) 1 month later, with 123 completing it. Ten (10) of those failed to complete the next interim questionnaire (T2) again 1 month later. Reasons reported for withdrawal at that point were “*did not think it was appropriate to be asked mental health questions*” from two participants and *“frustration at the inaccuracy of recording steps”*. Seventy (70) participants successfully completed the third interim questionnaire (T3) again 1 month later, and they were offered a £5 voucher to use at campus’ outlets, for completing all four waves. Finally, fifty-five (55) respondents participated in a follow-up survey (T4), 12 months after the completion of the trial (15 of those respondents reported still using the app at least occasionally). The recruitment process is summarised in Fig. 2 and the characteristics of the original sample are presented in Table 1.

**Table 1:** Sample Characteristics

	Baseline (T0) ***n*** = 148	
	Mean/ N (%)	SD
**Age in years**	38.19	9.42
**Gender: Male**	25 (16.9)	–
**Ethnic Group: White/ Caucasian**	129 (87.2)	–
**Ethnic Group: Black/African/Caribbean/Black British**	1 (0.7)	–
**Ethnic Group: Asian/ Asian British**	11 (7.4)	–
**Ethnic Group: Other**	7 (4.7)	–
**Operating System IoS**	89 (58.9)	–
**Highest level of education: Highschool**	8 (5.4)	–
**Highest level of education: College - A levels**	26 (17.6)	–
**Highest level of education: University - graduate**	61 (41.2)	–
**Highest level of education: University -postgrad**	35 (23.6)	–
**Highest level of education: University doctoral**	18 (12.2)	–
**Total Physical Activity** (MET)^a^	1823.72	3604.92
**Total Physical Activity (log x + 1)**	2.93	0.57
**Sleep Quality (PSQI) Total score**	6.8	2.31
**Life Satisfaction**	7.86	1.45
**Positive Affect**	3.46	0.79
**Negative Affect**	1.89	0.74

^a^Energy expenditure was measured in metabolic equivalents expended per week. *MET* Metabolic Equivalent Task

#### Power analysis

A post hoc power analysis for repeated measures analysis (within factors) was conducted with GPower 3.1 [16]. A group size of *n* = 62 was required to detect an effect size of d = 0.30 (f2 = 0.15 [9];) with statistical power of 0.80 (two-sided type one error level *p* = 0.05, assuming repeated measures correlation of r = 0.50 and 4 measurement times).

### Measures

#### Self-reported physical activity

The primary outcome variable was the total self-reported physical activity measured in metabolic equivalent of task (MET). It was assessed with questions from the short version of the International Physical Activity Questionnaire (IPAQ [12];) referring to the last 7 days prior to the questionnaire completion. The questions asked on how many days during the last 7 days and for how long on average participants engaged in (1) bicycling for transportation, (2) walking for transportation, (3) walking for leisure and recreation, (4) vigorous physical activity as well as (5) moderate physical activity for recreation, sport, exercise or leisure. A question on sedentary behaviour was not used. To transform physical activity into metabolic equivalents of task (MET), bicycling for transportation was weighted by 6.0, walking for transportation by 3.3, walking for leisure by 3.3, vigorous physical activity for leisure by 8.0, and moderate physical activity for leisure by 4.0 [12].

#### Device-measured physical activity

As secondary outcome variable, we used device-measured physical activity. Specifically, we used the daily step count as obtained by the accelerometer sensors in participants’ smartphones. That data was provided by Sweatcoin for each participant and included information from 1 month before the app was downloaded through to the end of the trial. We were only able to collect that data for iPhone users (*n* = 35 out of 70 who completed the three-month trial) due to the data being collated using Apple HealthKit (which allowed for measures prior to the app download to be captured). The device-measured step count was not used as primary outcome because it has not been validated for research purposes and it was only available for iOS users. To align with the self-reported physical activity, we calculated the mean daily step count for the seven-day period immediately prior to the date the participant had completed each questionnaire, including the baseline (T0). The self-reported physical activity and device-measured physical activity with the Apple HealthKit were significantly correlated at baseline T0 (*r* = 0.51, *p* = 0.001), T1 (*r* = 0.39, *p* < 0.05), and T2 (*r =* 0.37, *p* < 0.05) but not in T3 (*r* = 0.21, *p* > 0.05). It was not possible to gather participants’ daily step-count via the app in the follow-up period, T4. Participants were invited to manually download and share their Apple HealthKit step count history at the end of the follow-up questionnaire. However, only 9 participants provided their data, so no analyses of device-measured physical activity took place in T4.

#### Additional secondary outcomes

We measured three different components of SWB; namely life satisfaction, positive affect, and negative affect. Life satisfaction was measured with the three items: “Overall, how satisfied are you with your life nowadays”; “Overall, to what extent do you feel that the things you do in your life are worthwhile?”; and “Overall, how happy did you feel yesterday?” All items were answered originally on an eleven-points scale from 0 (*not at all*) to 10 (*completely*) and were subsequently recoded from 1 to 11. A single-item scale was calculated as the mean of the three items (Cronbach *α* = .84). Life satisfaction was not measured in the follow-up survey (T4). Positive and negative affect were each assessed with the ten items of the PANAS Scale [51]. The participants were asked to indicate the extent to which each of the 20 emotion-related adjectives described their current emotional state on a 5-point scale ranging from 0 (not at all) to 4 (to a large extent). The Positive and Negative Affect scores were computed as mean scores of the respective items, with Cronbach alphas of the two scales being *α* = .89 and .78, respectively. Finally, sleep quality was measured with the global score of Pittsburgh Sleep Quality Index (PSQI) [5] with higher values representing lower sleep quality.

### Analysis

To test our hypotheses, we ran mixed effects models for repeated measures with SPSS Statistics software (Version 25) using z-standardized values of the scales and measures. Standardization was conducted using the overall mean and standard deviation across all measurement time-points. The tested models allowed us to account for within- subjects (random effects) and between-subjects (fixed effects) variance [48]. We examined differences in physical activity, SWB and sleep quality before and after 3 months of consecutive use of the Sweatcoin application as well as 12 months after the opportunity to exchange Sweatcoins on campus had ceased (15 months after the beginning of the trial). Moreover, we ran correlation analyses for the device-measured and subjectively measured physical activity as well as for the examined outcome variables (i.e., physical activity, SWB, and sleep quality).

According to Cohen [9] an effect-size of d = 0.20 was considered small, d = 0.50 medium, and d = 0.80 large.

## Results

### Changes in physical activity, subjective well-being, and sleep quality

**Fig. 3:**
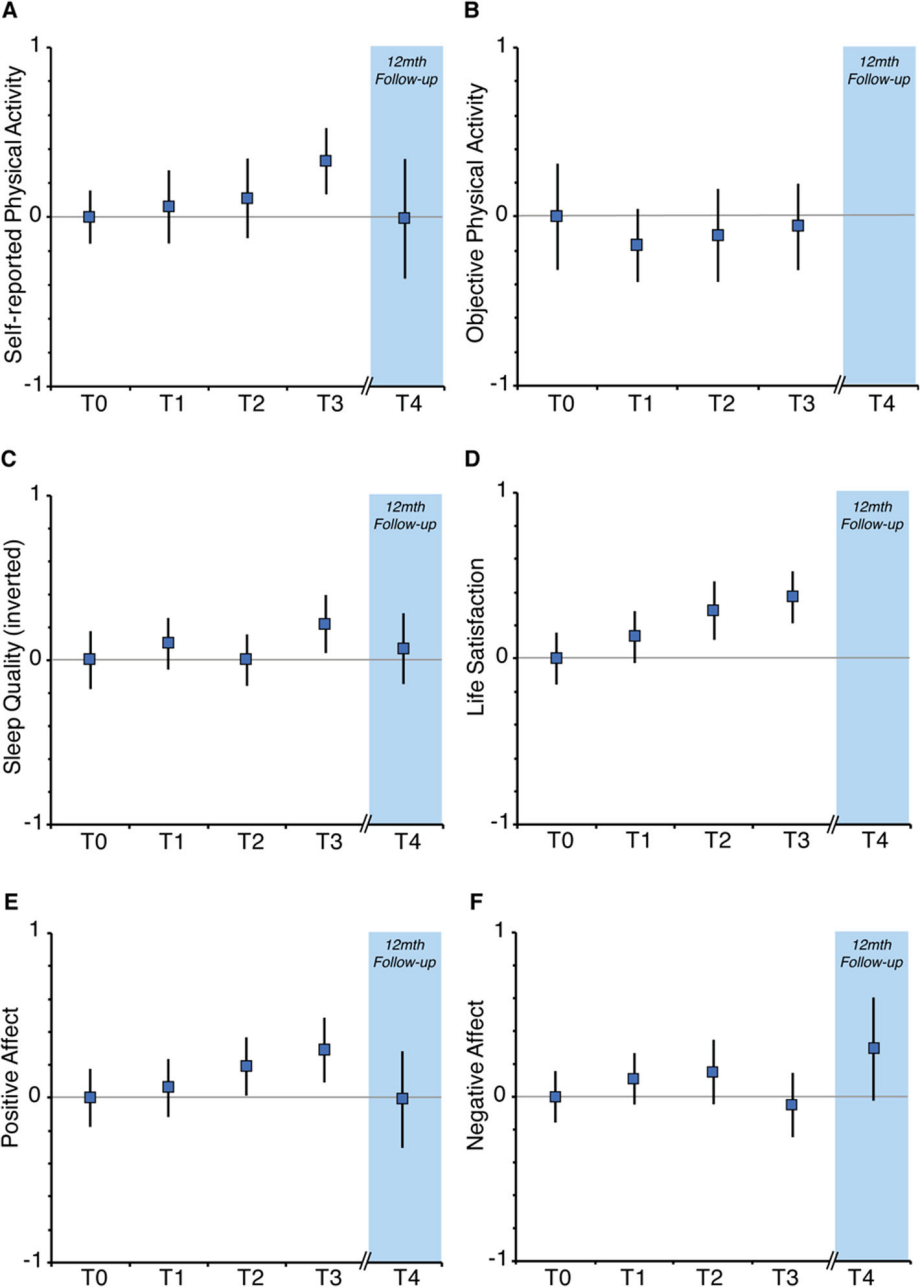
Results from the primary and secondary outcome measures, standardised as z-scores over the time periods T0 (baseline), T1 (after 1 month), T2 (after 2 months), T3 (after 3 months) and T4 (follow-up, 12 months after T3). Error bars indicate 95% confidence intervals. The scores of Physical Activity represent metabolic equivalent of task (MET). The scores of Sleep Quality are inverted in the Figure (such that higher values indicate better sleep) to improve readability

The results are illustrated in Fig. 3 showing standardized scores.

#### Primary outcome

The mean self-reported physical activity measured in metabolic equivalents (MET) increased from T0 (*M* = -0.12) to T3 (*M* = 0.22), that is, during the 3 months of Sweatcoin app use reflecting a small to medium effect size (*d* = 0.34). The increase was not sustained 12 months after the end of the trial (T4 = -0.13).

#### Secondary outcome

Device-measured physical activity (Apple HealthKit), remained largely on the same level between T0 (*M* = 0.07) and T3 (*M* = 0.02).

#### Additional secondary outcomes

***Subjective well-being (SWB)*** Life satisfaction showed a gradual increase between T0 (*M* = -0.18) and T3 (*M* = 0.13) reflecting a small to medium effect size (*d* = 0.31) (no measurement at 1-year follow-up available). Similarly, positive affect (T0: *M* = -0.10) increased after 2 months of consecutive use of the Sweatcoin app (T2: *M* = 0.09) and even further after 3 months (T3: *M* = 0.19) reflecting a small to medium effect size (*d* = 0.29). The increase was not sustained 12 months after the opportunity to exchange Sweatcoins on Campus had ceased (T4: *M* = -0.11). Negative affect remained largely on a similar level between T0 (*M* = 0.07) and T3 (*M* = -0.12), however there was an increase at T4 (*M* = 0.23).

***Sleep quality*** The score of sleep quality decreased from T0 (*M* = 0.09) to T3 (*M* = -0.13) indicating a small improvement in sleep quality (d = 0.22) as lower values in PSQI represented better sleep quality. The effect was not sustained at T4 (*M* = 0.02).

### Intention to treat (ITT) analysis

ITT analysis was conducted to examine whether respondents’ drop out affected the results [35]. ITT analysis allowed us to analyse the data of all those who originally participated in the trial regardless of whether they completed it and test whether the original analyses’ results were subject to selection bias [1]. What the ITT analysis involved was the repetition of the main analyses using the data of the 148 respondents who participated in T0 replacing the missing values that occurred due to drop-out at subsequent measurements with the scores obtained at the baseline (T0). The results remained the same in terms of statistical significance and effect-size (Supplementary Table 2).

## Discussion

Our results suggest that the use of the ‘rewards-for-exercise’ application Sweatcoin is associated with a short-term increase in self-reported physical activity, life satisfaction, positive affect, and sleep quality while it was not associated with changes in device-measured physical activity. The findings are in line with previous studies showing that self-monitoring and rewarding applications contribute to increased exercise levels [19, 40, 45], SWB [3, 6, 8, 18, 31, 39, 42], and sleep quality [26]. Several mechanistic pathways have been suggested by which physical activity may improve sleep quality which involve changes in circadian regulation, increased build-up of sleep pressure, and decrease in anxiety symptoms related to improved ability to relax [26, 47]. Further, physical activity may increase SWB by increasing positive affect and self-affirmation related to increased physical fitness [23]. However, the likely effect was not sustained 12 months after the opportunity to exchange Sweatcoins at the in-app ‘local marketplace’ had ceased.

Further, our findings agree with existing studies showing that people are more likely to change their physical activity patterns, if they are provided with tangible and directly accessible rewards [28]. The fact that Sweatcoin users were able to access a ‘local marketplace’ across the university campus may have served the purpose of making the application’s rewarding system more effective; participants could easily enjoy the benefits of their increased physical activity by simply exchanging sweatcoins with products they would buy on a routine basis while at work. Additionally, the observation that changes in physical activity vanished a year after the end of the trial suggests even further that the direct access to tangible rewards is crucial for the effectiveness of behaviour change interventions. Moreover, this finding is also consistent with previous research showing that changes in behaviour are not sustained when financial incentives disappear [20, 33, 49].

Our findings should be read in the light of certain limitations. First, our study lacks a control group and randomization to intervention and therefore cannot be used to draw causal conclusions. Effects on outcome measures could be attributed to factors relevant to the seasons of measurement (i.e. T0/baseline was in February while T3 was in May). However, we show that in the follow-up assessment (T4), which was also conducted in May but 12 months later, all the effects had vanished. As the change in behaviour was contingent on the presence of reward the findings suggest that the effects were not due to the season of testing. Second, with the current study we were not able to disentangle the effects of the multiple elements of the Sweatcoin application (i.e. networking opportunities, self-regulation, maintenance of a profile). Future research should investigate the role of these factors and the effectiveness of different incentive structures [49]. Third, regarding device-measured physical activity, we were only able to measure steps rather than other types of physical activity and only when the phone was carried. This indicates that the device-measured physical activity in our study was likely to have underestimated respondents’ actual levels of physical activity. Future studies would need to use more accurate measurement tools. Moreover, device-measured physical activity was only available for around half of the participants using the Apple HealthKit, which uses the accelerometer of the iPhone. This reduced the available data and has made it difficult to draw accurate conclusions due to lack of statistical power. Fourth, the participants, who were screened to be included in the study, were self-selected and might have been more motivated to change their behaviour. To estimate the overall effectiveness of the intervention in workplace settings it will be necessary to conduct a randomized trial with non-self-selected participants. Fifth, there was substantial attrition between baseline and the end of the 3-month trial. However, ITT analysis showed that selective sample dropout did not explain the results. Finally, participants were not blind with regard to the intervention they received and therefore placebo effects could explain the results.

## Conclusions

This study suggests that mobile incentives-for-exercise applications might increase physical activity levels, positive affect, ratings of life satisfaction, and sleep quality, at least in the short term. Thus, the possibility to receive tangible rewards through everyday routines and across a local and accessible market may be effective in encouraging physical activity in a working environment and encourages employers and labour market institutions to further invest in such interventions. As the study lacked a control group, no firm conclusions about causal effects are possible. Future research applying randomized controlled designs is needed to confirm the findings. If such research confirmed the effectiveness, the intervention could be used on a broad scale in workplace settings to increase physical activity of the work force at least for as long as the intervention is continued.

## Supplementary Information

**Additional file 1: Table S1.** List of products available from retail outlets in the University campus offered as rewards on the in-app local marketplace. **Table S2.** Intention to Treat Analysis’ Results.

## Data Availability

The datasets used and/or analysed during the current study are available from the corresponding author on reasonable request.

## Abbreviations

BSREC: Biomedical and Scientific Research Ethics Committee
GPPAQ: General Practice Physical Activity Questionnaire
GPS: Global Positioning System
IPAQ: International Physical Activity Questionnaire
ITT: Intention to Treat
MET: Metabolic Equivalent of Task
mHealth: Mobile Health
PANAS: Positive and Negative Affect Scale
PSQI: Pittsburgh Sleep Quality Index
SWB: Subjective Wellbeing

## Footnotes

1 Participants were excluded because they did not meet one or more criteria.
