# Supplementary material for: Can a ‘rewards-for-exercise app’ increase physical activity, subjective well-being and sleep quality? An open-label single-arm trial among university staff with low to moderate physical activity levels
Source: BMC Public Health. 2021 Apr 23;21:782. doi: 10.1186/s12889-021-10794-w (PMC8063391; doi:10.1186/s12889-021-10794-w)
Supplement: Supplementary file 1 — Additional file 1: Table S1. List of products available from retail outlets in the University campus offered as rewards on the in-app local marketplace. Table S2. Intention to Treat Analysis’ Results. [file 12889_2021_10794_MOESM1_ESM.docx]

***Supplementary Table 1. List of products available from retail outlets in the University campus offered as rewards on the in-app local marketplace.***

| **Product** | **Total number redeemed** | **Retail Value** | **Price in Sweatcoins** |
| --- | --- | --- | --- |
| Coffee | 41 | £2.40 | 40 |
| Sandwich meal deal | 13 | £4.50 | 60 |
| Hot lunch | 13 | £6.00 | 70 |
| Cooked breakfast | 10 | £3.50 | 70 |
| Hot and cold drinks | 10 | £3.50 | 60 |
| Sandwiches | 5 | £3.50 | 50 |
| Meal for 2 at campus-based restaurant | 12 | £15.00 | 100 |
| Notebook | 3 | £4.99 | 50 |
| Keyring | 2 | £4.45 | 20 |
| Drinks bottle | 1 | £6.90 | 20 |
| Leather bookmark | 3 | £2.50 | 20 |
| University branded hoodie | 1 | £21.00 | 180 |
| Moleskin notebook | 1 | £20.00 | 150 |
| China mug | 2 | £8.00 | 90 |
| Pen set | 2 | £10.00 | 90 |
| Arts Centre cinema tickets | 7 | £18.00 | 150 |
| Arts Centre theatre tickets | 5 | £36.00 | 200 |
| 30-day gym membership | 1 | £25.00 | 180 |
| 6-month gym membership | 4 | £55.00 | 250 |

|  | **M** | **SE** | **95% CI** | **B** | **SE** | **P** |
| --- | --- | --- | --- | --- | --- | --- |
| **Self-reported Physical Activity** |  |  |  |  |  |  |
| *Baseline (T0)* | -0.12 | 0.08 | -0.28, 0.04 |  |  |  |
| *T1* | -0.08 | 0.09 | -0.26, 0.13 | 0.04 | 0.10 | 0.694 |
| *T2* | -0.04 | 0.11 | -0.25, 0.17 | 0.08 | 0.11 | 0.494 |
| *T3* | 0.20 | 0.09 | 0.03, 0.11 | 0.32 | 0.10 | 0.002 |
| *T4* | -0.14 | 0.15 | -0.28, 0.04 | -0.02 | 0.16 | 0.902 |
| **Objective Physical Activity** |  |  |  |  |  |  |
| *Baseline (T0)* | 0.07 | 0.16 | -0.24, 0.38 |  |  |  |
| *T1* | -0.10 | 0.16 | -0.42, 0.22 | -0.17 | 0.11 | 0.133 |
| *T2* | -0.03 | 0.17 | -0.37, 0.32 | -0.10 | 0.13 | 0.467 |
| *T3* | 0.02 | 0.17 | -0.32, 0.36 | -0.05 | 0.13 | 0.709 |
| **Life Satisfaction** |  |  |  |  |  |  |
| *Baseline (T0)* | -0.18 | 0.09 | -0.35, -0.01 |  |  |  |
| *T1* | -0.02 | 0.09 | -0.19, 0.16 | 0.16 | 0.08 | 0.039 |
| *T2* | 0.05 | 0.10 | -0.15, 0.24 | 0.23 | 0.09 | 0.012 |
| *T3* | 0.14 | 0.09 | -0.05, 0.32 | 0.32 | 0.08 | 0.000 |
| **Positive Affect** |  |  |  |  |  |  |
| *Baseline (T0)* | -0.10 | 0.09 | -0.27, 0.07 |  |  |  |
| *T1* | -0.02 | 0.09 | -0.20, 0.15 | 0.08 | 0.09 | 0.395 |
| *T2* | 0.09 | 0.09 | -0.08, 0.26 | 0.19 | 0.09 | 0.033 |
| *T3* | 0.19 | 0.09 | 0.01, 0.38 | 0.29 | 0.10 | 0.002 |
| *T4* | -0.11 | 0.15 | -0.40, 0.18 | -0.01 | 0.15 | 0.923 |
| **Negative Affect** |  |  |  |  |  |  |
| *Baseline (T0)* | -0.07 | 0.08 | -0.22, 0.09 |  |  |  |
| *T1* | 0.02 | 0.09 | -0.15, 0.18 | 0.08 | 0.08 | 0.311 |
| *T2* | 0.06 | 0.09 | -0.13, 0.26 | 0.13 | 0.09 | 0.18 |
| *T3* | -0.14 | 0.10 | -0.34, 0.07 | -0.07 | 0.10 | 0.463 |
| *T4* | 0.22 | 0.16 | -0.10, 0.53 | 0.28 | 0.16 | 0.076 |
| **Sleep Quality** |  |  |  |  |  |  |
| *Baseline (T0)* | 0.09 | 0.09 | -0.08, 0.26 |  |  |  |
| *T1* | 0.00 | 0.08 | -0.17, 0.16 | -0.09 | 0.07 | 0.222 |
| *T2* | 0.09 | 0.09 | -0.09, 0.26 | 0.00 | 0.08 | 0.983 |
| *T3* | -0.12 | 0.10 | -0.31, 0.06 | -0.21 | 0.08 | 0.014 |
| *T4* | 0.02 | 0.12 | -0.21, 0.25 | -0.07 | 0.11 | 0.517 |

***Supplementary Table 2. Intention to Treat Analysis' Results***
